# Antiviral therapy can effectively suppress irAEs in HBV positive hepatocellular carcinoma treated with ICIs: validation based on multi machine learning

**DOI:** 10.3389/fimmu.2024.1516524

**Published:** 2025-01-27

**Authors:** Shuxian Pan, Zibing Wang

**Affiliations:** Department of Immunotherapy, The Affiliated Cancer Hospital of Zhengzhou University & Henan Cancer Hospital, Zhengzhou, China

**Keywords:** hepatocellular carcinoma, immunotherapy, ICIS, irAEs, machine learning

## Abstract

**Background:**

Immune checkpoint inhibitors have proven efficacy against hepatitis B-virus positive hepatocellular. However, Immunotherapy-related adverse reactions are still a major challenge faced by tumor immunotherapy, so it is urgent to establish new methods to effectively predict immunotherapy-related adverse reactions.

**Objective:**

Multi-machine learning model were constructed to screen the risk factors for irAEs in ICIs for the treatment of HBV-related hepatocellular and build a prediction model for the occurrence of clinical IRAEs.

**Methods:**

Data from 274 hepatitis B virus positive tumor patients who received PD-1 or/and CTLA4 inhibitor treatment and had immune cell detection results were collected from Henan Cancer Hospital for retrospective analysis. Models were established using Lasso, RSF (RandomForest), and xgBoost, with ten-fold cross-validation and resampling methods used to ensure model reliability. The impact of influencing factors on irAEs (immune-related adverse events) was validated using Decision Curve Analysis (DCA). Both uni/multivariable analysis were accomplished by Chi-square/Fisher’s exact tests. The accuracy of the model is verified in the DCA curve.

**Results:**

A total of 274 HBV-related liver cancer patients were enrolled in the study. Predictive models were constructed using three machine learning algorithms to analyze and statistically evaluate clinical characteristics, including immune cell data. The accuracy of the Lasso regression model was 0.864, XGBoost achieved 0.903, and RandomForest reached 0.961. Resampling internal validation revealed that RandomForest had the highest recall rate (AUC = 0.892). Based on machine learning-selected indicators, antiviral therapy and The HBV DNA copy number showed a significant correlation with both the occurrence and severity of irAEs. Antiviral therapy notably reduced the incidence of IRAEs and may modulate these events through regulation of B cells. The DCA model also demonstrated strong predictive performance. Effective control of viral load through antiviral therapy significantly mitigates the occurrence of irAEs.

**Conclusion:**

ICIs show therapeutic potential in the treatment of HBV-HCC. Following antiviral therapy, the incidence of severe irAEs decreases. Even in cases where viral load control is incomplete, continuous antiviral treatment can still mitigate the occurrence of irAEs.

## Introduction

Hepatocellular carcinoma, distinguished by its high incidence and fatality rates, is the sixth most common of cancer-related deaths worldwide (1). Primary liver cancer is mainly hepatocellular carcinoma, accounting for more than 70% (2). Research shows that high risk factors related to liver cancer are related to multiple viral infections, the main ones being hepatitis B virus and hepatitis C virus (3). For patients with liver cancer in early clinical stages, clinical treatment methods mainly include surgical resection, local therapeutic intervention, liver allotransplantation, etc (4, 5). However, for patients with high clinical stages of liver cancer, current clinical intervention methods still cannot effectively control recurrence or metastasis within 5 years (4). Currently, the preferred clinical treatment for advanced liver cancer is targeted therapy based on anti-tumor angiogenesis-related drugs, but the clinical benefits are still poor (6).

T cells’ intrinsic negative immune regulators, such as CTLA-4, PD-1, and their ligands, can be blocked by immune checkpoint inhibitors (ICIs), which enhance T cell cytotoxicity and augment the antitumor activity of T lymphocytes (7). Studies have shown that ICIs have provided significant benefits in treating various cancers, including lung cancer, melanoma, renal cell carcinoma, and head and neck tumors (8–11). In individuals with early-stage hepatocellular carcinoma, PD-1 inhibitors like nivolumab and pembrolizumab have shown substantial clinical efficacy, markedly improving both overall survival and disease-free survival rates (12–14). The combination of anti-PD-1 antibodies and anti-angiogenic therapy, such as bevacizumab, has shown even greater clinical benefits in patients with advanced liver cancer (15, 16). Moreover, the therapeutic potential of cabozantinib in accompanied with pembrolizumab for the remedy of advanced hepatocellular carcinoma is actively being assessed (17). Despite the remarkable success of ICIs in advanced liver cancer, predictive factors for their clinical efficacy remain limited, with microsatellite instability, gut microbiota and TMB (tumor mutation burden) being among the few identified (18–20).

irAEs are a manifestation of the inherent limitations of immune tolerance, primarily induced by immune checkpoint inhibitors (ICIs) that trigger the production of auto-antibodies and pathogenic antibodies (21–24). These irAEs have the potential to impact any organ system and are categorized into five distinct grades according to their severity (9, 25). Clinically, patients receiving ICI therapy require frequent monitoring to mitigate the risk of irAEs (26, 27). Research indicates that irAEs are intricately linked to the function of immune checkpoint inhibitors (ICIs) in preserving immune homeostasis. Multiple potential mechanisms have been suggested, including T cell activation against self-antigens, the production of auto-antibodies and pro-inflammatory cytokines, as well as increased complement activation targeting self-antigens (7, 28). The mechanism of occurrence of immune-related adverse events determines the specificity of their systemic pathogenesis, including inflammatory arthritis, Sjögren’s disease, vasculitis, joint pain, or tendinopathy (29–31). In severe cases, bone marrow suppression may even occur (32). Because of this, the occurrence of immune-related adverse events seriously affects the clinical treatment of cancer patients and has become an important issue that must be addressed (33).

Recent studies have revealed that severe irAEs can interrupt cancer patients’ immunotherapy, potentially hindering the clinical benefits of these treatments (28). Although there has been some research on the management of irAEs, identifying clinical indicators and methods to predict or mitigate irAEs remains an urgent need. In this study, we analyzed clinical data from liver cancer patients undergoing immunotherapy to assess the risk factors and associated indicators of irAEs.

## Methods

### Enrollment of patients

This retrospective study encompassed patients diagnosed with liver cancer at the Affiliated Cancer Hospital of Zhengzhou University from January 2019 to February 2024, the process of including cases in the study is shown in the **Figure 7**. Diagnosis was based on clinical pathology and imaging in accordance with the criteria set by the American Association for the Study of Liver Diseases (AASLD), including laboratory-confirmed positive HBV DNA serology, with all patients having undergone at least one PD-1 inhibitor treatment. Clinical data were meticulously gathered through manual examination of patient records and pertinent test outcomes. Brought into criteria were: 1. Patients older than 18. 2. Positive laboratory results for HBV DNA. 3. Eastern Cooperative Oncology Group performance status (ECOG PS) scores ranging 0 - 2, with at least one measurable lesion per the Response Evaluation Criteria in Solid Tumors (RECIST) 1.1 guidelines. The efficacy of immunotherapy was evaluated based on RECIST 1.1 standards, categorizing outcomes as complete response (CR), partial response (PR), stable disease (SD), or progressive disease (PD). 4. The severity of immune-related adverse events (irAEs) was classified by the Common Terminology Criteria for Adverse Events (CTCAE 5.0) established by the U.S. National Cancer Institute. 5. Patients on antiviral therapy were included if they had received such treatment prior to or concurrently with PD-1 inhibitors. Among the included cases, 20 patients unreceived anti-viral treatment during ICIs, 44 exhibited poor compliance and ceased antiviral therapy before hospitalization, 18 discontinued due to financial difficulties, and 7 self-discontinued antiviral therapy prior to immunotherapy. 6. Laboratory evaluations encompassed peripheral blood immune cell assays, treatment protocols, clinical outcomes, and related biochemical results. HBV reactivation was defined per the 2018 AASLD hepatitis B guidelines, meeting at least one of the following criteria: (i) virus DNA increase of ≥ 2 log (100-fold) versus baseline; (ii) DNA increase ≥ 3 log (1,000) IU/mL (for patients non-detectable previously serum virus DNA, recognizing the potential for fluctuations in HBV DNA levels); or (iii) if baseline levels were unavailable, virus DNA increase ≥ 4 log (10,000) IU/mL (34). The albumin/bilirubin (ALBI) grade was calculated using the formula: (0.66 × log10 bilirubin) + (−0.085 × albumin), with bilirubin measured in μmol/L and albumin in g/L. The grading criteria are defined as follows: Grade 1, ALBI ≤ −2.60; Grade 2, −2.60 < ALBI ≤ −1.39; and Grade 3, ALBI > −1.39 (35).

### Statistical analysis

The results of this study, along with the relevant statistical analyses, were completed by R language (version 4.4.0). Numerical variables that adhering normal distribution are expressed as mean ± standard, chi-square or Fisher’s exact test were utilized for analysis of categorical variables. Lasso (glmnet-4.1-8), RSF (randomForest-4.7), and XGBoost (xgBoost-2.1.3) were used to assess the importance of both categorical and numerical variables in predicting outcomes over the observation period. Rank-sum tests were used to evaluate differences in stratified data, and univariate analyses were performed using two-tailed t-tests. p <0.05 means statistically significant.

## Results

### The clinical baseline characteristics of enrolled patients

A total of 274 HBV-positive liver cancer patients who received ICIs treatment were enrolled in the research. clinical characteristics are summarized in **Supplementary Table 1**. All the enrolled samples, 191 were male (69.7%) and 83 were female (30.3%). Among male patients, 72% experienced grade 1-2 irAEs, while 28% of female patients experienced grade 1-2 irAEs. The cohort included 119 patients (43.4%) aged 60 and older, and 155 patients (56.6%) under 60. A total of 214 patients (78.1%) developed grade 1-2 irAEs, while 60 patients (21.9%) experienced grade 3-4 irAEs.

As shown in **Table 1**, 42.1% of patients aged 60 and older developed grade 1-2 irAEs, compared to 57.9% of patients under 60. Of the 184 patients who received antiviral treatment during PD-1 inhibitor therapy, 168 (78.5%) experienced grade 1-2 irAEs, and 16 (26.7%) experienced grade 3-4 irAEs. According to the RECIST evaluation, 7 patients achieved complete response (CR), 81 had partial response (PR), 128 had stable disease (SD), and 51 experienced progressive disease (PD). Among patients with PR and CR, 72 (77.4%) received antiviral treatment, compared to 21 (22.6%) who did not, with a significant difference (p = 0.014, **Supplementary Table 1**).

**Table 1:** Baseline information on clinical subgroups of patients with different grades of immune adverse events.

Name	Levels	G1-G2 (N=214)	G3-G4 (N=60)	p
Gender	female	60 (28%)	23 (38.3%)	.169
	male	154 (72%)	37 (61.7%)	
Age	<60	124 (57.9%)	31 (51.7%)	.472
	>=60	90 (42.1%)	29 (48.3%)	
DNA(HBV)	<500	153 (71.5%)	25 (41.7%)	<.001
	>=500	61 (28.5%)	35 (58.3%)	
Alcohol	No	108 (50.5%)	30 (50%)	1.000
	Yes	106 (49.5%)	30 (50%)	
Antivirus_therapy	Anti-virus	168 (78.5%)	16 (26.7%)	<.001
	No-antivirus	46 (21.5%)	44 (73.3%)	
Antivirus. drug		46 (21.5%)	39 (65%)	<.001
	Adefovir ester	6 (2.8%)	0 (0%)	
	Entecavir	155 (72.4%)	21 (35%)	
	Tenofovir	5 (2.3%)	0 (0%)	
	Tenofovir disoproxil	2 (0.9%)	0 (0%)	
Surgery	No	149 (69.6%)	39 (65%)	.600
	Yes	65 (30.4%)	21 (35%)	
Interventional_therapy	No	55 (25.7%)	20 (33.3%)	.313
	Yes	159 (74.3%)	40 (66.7%)	
Radiotherapy	No	178 (83.2%)	49 (81.7%)	.936
	Yes	36 (16.8%)	11 (18.3%)	
Tcellpercent	Mean ± SD	68.6 ± 11.7	71.0 ± 11.7	.163
CD8percent	Mean ± SD	25.5 ± 9.3	26.5 ± 10.7	.461
CD4percent	Mean ± SD	36.6 ± 10.7	38.8 ± 11.7	.166
NKcellpercent	Mean ± SD	18.9 ± 11.1	17.3 ± 11.2	.336
Bcellpercent	Mean ± SD	9.9 ± 6.8	8.3 ± 5.3	.056
Tregs	Mean ± SD	9.1 ± 2.4	9.1 ± 3.0	.969
PD1percent	Mean ± SD	8.6 ± 8.3	10.6 ± 9.3	.100
PD1CD3cellpercent	Mean ± SD	11.7 ± 11.0	14.8 ± 12.9	.070
PD1CD4cellpercent	Mean ± SD	11.8 ± 11.4	14.4 ± 12.9	.140
PD1CD8cellpercent	Mean ± SD	12.0 ± 12.7	15.5 ± 14.6	.075
lym	Mean ± SD	1476.8 ± 716.4	1442.9 ± 829.0	.759
Tcells	Mean ± SD	1015.8 ± 502.5	1076.7 ± 686.8	.531
CD4	Mean ± SD	543.4 ± 293.6	564.6 ± 373.9	.691
CD3CD8	Mean ± SD	368.4 ± 229.9	402.5 ± 355.4	.491
NKcells	Mean ± SD	296.9 ± 229.8	248.1 ± 209.2	.146
Bcells	Mean ± SD	149.6 ± 143.3	109.1 ± 78.3	.005
AFP	<400	127 (59.3%)	37 (61.7%)	.653
	>=400	43 (20.1%)	9 (15%)	
	>400	44 (20.6%)	14 (23.3%)	
TB	Mean ± SD	21.9 ± 13.4	28.8 ± 42.0	.217
Albumin	Mean ± SD	41.3 ± 5.9	39.3 ± 6.1	.023
ALBI	Mean ± SD	-2.7 ± 0.5	-2.4 ± 0.5	.006
ALBI score	Mean ± SD	1.5 ± 0.5	1.7 ± 0.5	.018
ECOG	Mean ± SD	1.0 ± 0.6	1.0 ± 0.7	.852
Child-Pugh	A	169 (79%)	49 (81.7%)	.782
	B	45 (21%)	11 (18.3%)	
BCLC		16 (7.5%)	7 (11.7%)	.496
	A	13 (6.1%)	6 (10%)	
	B	51 (23.8%)	13 (21.7%)	
	C	134 (62.6%)	34 (56.7%)	
ALT	Mean ± SD	55.8 ± 54.5	71.2 ± 75.3	.143
AST	Mean ± SD	70.9 ± 78.2	80.0 ± 104.3	.532
Tumor diameter		30 (14%)	8 (13.3%)	.808
	<3	63 (29.4%)	18 (30%)	
	>5	82 (38.3%)	26 (43.3%)	
	3~5	39 (18.2%)	8 (13.3%)	
Liver cirrhosis	No	73 (34.1%)	22 (36.7%)	.831
	Yes	141 (65.9%)	38 (63.3%)	
Vascular invasion	No	155 (72.4%)	44 (73.3%)	1.000
	Yes	59 (27.6%)	16 (26.7%)	
PD-1 inhibitor	Camrelizumab	150 (70.1%)	40 (66.7%)	.354
	Camrelizumab+Sintilimab	5 (2.3%)	1 (1.7%)	
	Camrelizumab+Tislelizumab	3 (1.4%)	1 (1.7%)	
	Nivolumab	2 (0.9%)	3 (5%)	
	Pembrolizumab	1 (0.5%)	0 (0%)	
	Pembrolizumab+Toripalimab	0 (0%)	1 (1.7%)	
	Sintilimab	26 (12.1%)	7 (11.7%)	
	Tislelizumab	25 (11.7%)	7 (11.7%)	
	Toripalimab+Sintilimab	2 (0.9%)	0 (0%)	
Outcome		4 (1.9%)	3 (5%)	.107
	CR	7 (3.3%)	0 (0%)	
	PD	44 (20.6%)	7 (11.7%)	
	PR	65 (30.4%)	16 (26.7%)	
	SD	94 (43.9%)	34 (56.7%)	

Patients with grade 1-2 irAEs had a lower proportion of HBV DNA levels above 500 IU/mL compared to those with grade 3-4 irAEs (p < 0.001). Additionally, patients who received antiviral therapy had a significantly higher proportion of irAEs (p < 0.001), with notably elevated absolute B cell counts (p = 0.005) and significantly lower ALBI scores (p = 0.006). However, no significant differences were observed in other immune cell proportions and absolute counts, tumor size, ECOG scores, alpha-fetoprotein levels, treatment regimens, vascular invasion, or liver function across the different grades of irAEs. **Supplementary Table 2** demonstrates that liver cancer patients who received antiviral therapy had a significantly higher proportion of clinical benefit from immunotherapy (p = 0.014), **Supplementary Tables 3** and **4** present the statistics of the occurrence of immune-related adverse reactions in different organs and the effects of different antiviral drugs on the efficacy of immunotherapy, respectively.

### Biomarkers selection for prediction of irAEs

**Figure 1:**
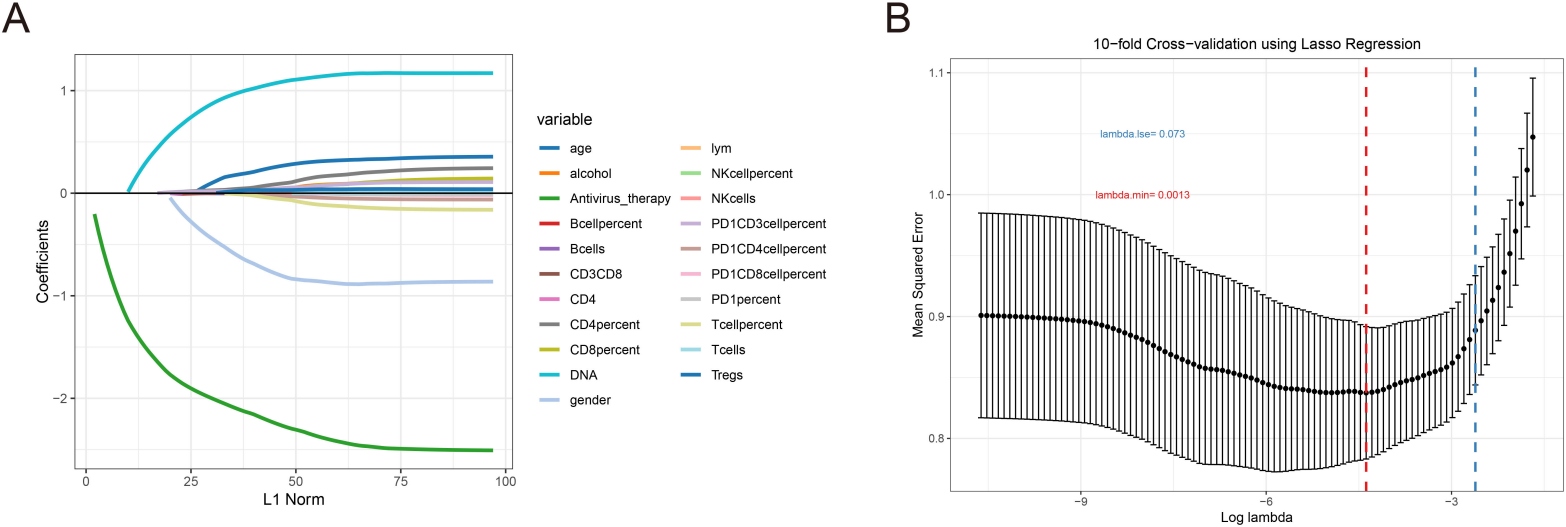
LASSO coefficient was used to analyze the risk factors of immune-related adverse events. **(A)** Lasso regression ten-fold cross validation curve. **(B)** In the LASSO model, the non-zero coefficient characteristic curve is extracted from the log (A) series. The vertical dashed lines are drawn at the minimum mean square error (λ = 0.0013) and the minimum distance standard error (λ = 0.073).

**Figure 2:**
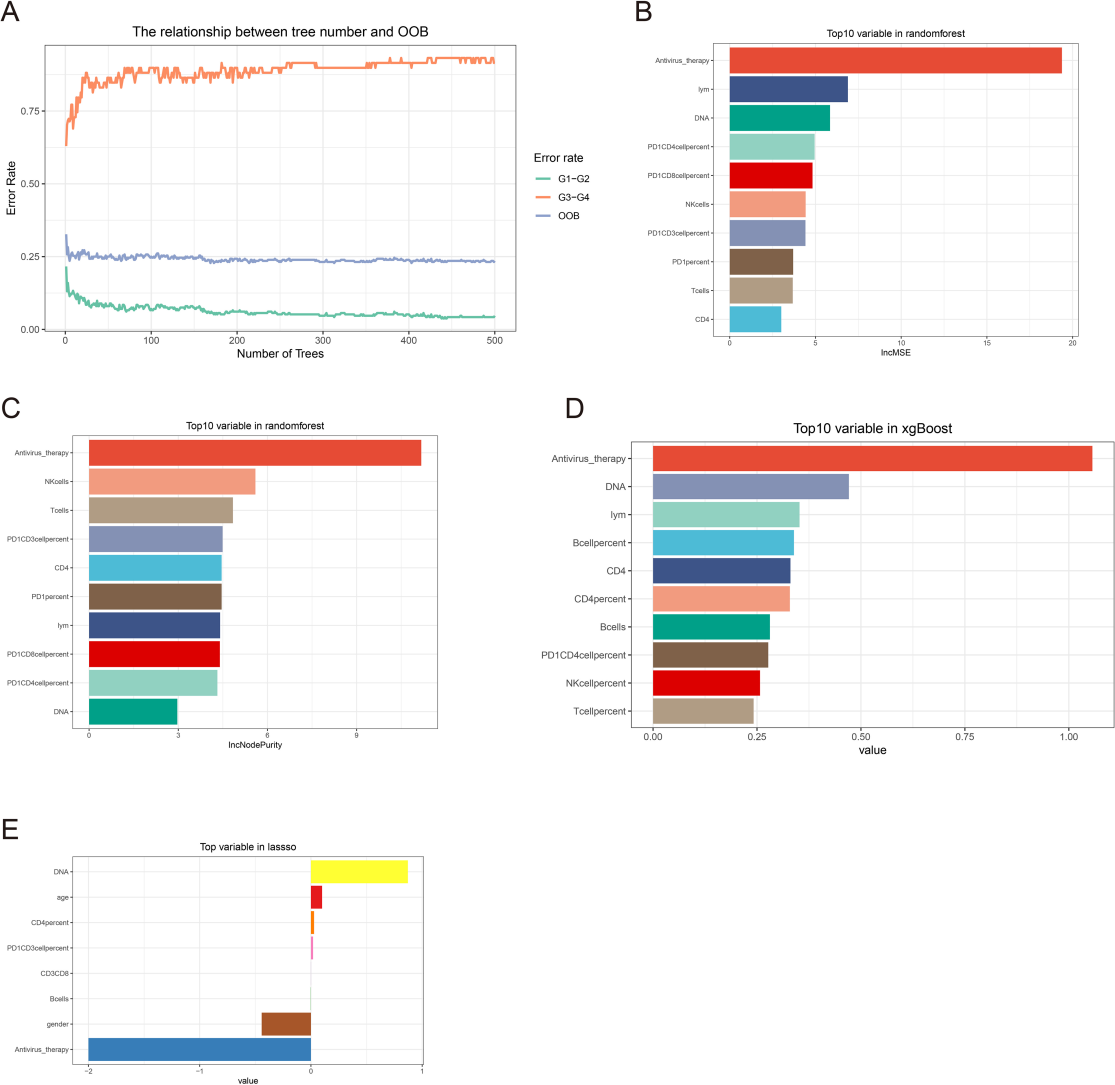
Machine learning feature screening. **(A)** Random forest graph model error curve. **(B, C)** Random forest ranking of clinical features by importance. **(D)** Xgboost clinical feature importance ranking. **(E)** Clinical characteristics of non-zero coefficients in lasso regression.

To identify clinical indicators associated with irAEs, we conducted a lasso regression analysis on the selected clinical parameters, utilizing ten-fold cross-validation. The results from the lasso regression are displayed in **Figure 1A**, showing the distribution of clinical characteristics after applying the lasso regression model. Cross-validation parameters were optimized using the minimum lambda value (lambda min), and both the optimal lambda min and lambda standard error (lambda se) were used to generate the ten-fold cross-validation curve (**Figure 1B**). The minimum standard value was identified through cross-validation, and the corresponding ten-fold cross-validation curve was plotted (**Figure 1B**). As a result, we identified seven clinical parameters with non-zero coefficients (**Figure 2E**). Univariate and multivariate logistic regression analyses further confirmed that antiviral therapy and HBV DNA levels were independent risk factors for the occurrence of irAEs (**Table 2**).

**Table 2:** Univariate and multivariate logistic regression with non-zero coefficients in lasso regression.

Dependent:level		G1-G2 (n=213)	G3-G4 (N=59)	OR (univariable)	OR (multivariable)
Gender	Female	60 (28.2%)	23 (39%)		
	Male	153 (71.8%)	36 (61%)	0.61 (0.34-1.12, p=.112)	0.52 (0.25-1.07, p=.077)
Age	<60	123 (57.5%)	30 (50.8%)		
	>=60	90 (42.3%)	29 (49.2%)	1.32 (0.74-2.36, p=.345)	
DNA	<500	151 (70.9%)	25 (42.4%)		
	>=500	62 (29.1%)	34 (57.6%)	3.31 (1.83-6.01, p<.001)	
Antivirus_therapy	Anti-virus	167 (78.4%)	16 (27.1%)		
	No-antivirus	46 (21.6%)	43 (72.9%)	9.76 (5.04-18.88, p<.001)	8.21 (4.12-16.37, p<.001)
CD4percent	Mean±SD	36.6±10.8	38.5±11.5	1.02 (0.99-1.04, p=.233)	
PD1CD3cellpercent	Mean±SD	11.8±11.1	14.7±12.9	1.02 (1.00-1.05, p=.094)	1.03 (1.00-1.06, p=.047)
CD3CD8	Mean±SD	371.8±235.6	399.5±353.0	1.00 (1.00-1.00, p=.479)	
Bcells	Mean±SD	153.3±146.0	107.6±78.5	1.00 (0.99-1.00, p=.024)	1.00 (0.99-1.00, p=.102)

### Multi-machine learning model construction

**Figure 3:**
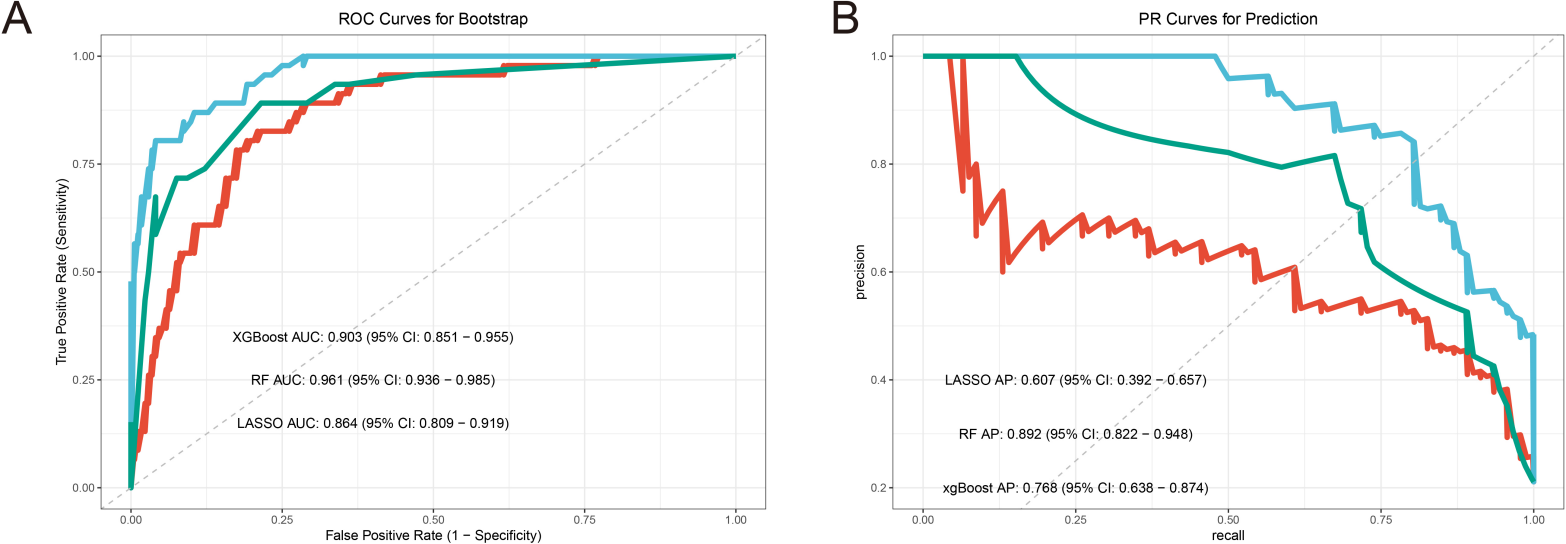
Machine learning model performance analysis. **(A)** Boostrap resampling verifies the accuracy AUC curve of the machine learning model. **(B)** ROC curve of bootstrap resampling to verify the accuracy of machine learning model.

**Figure 4:**
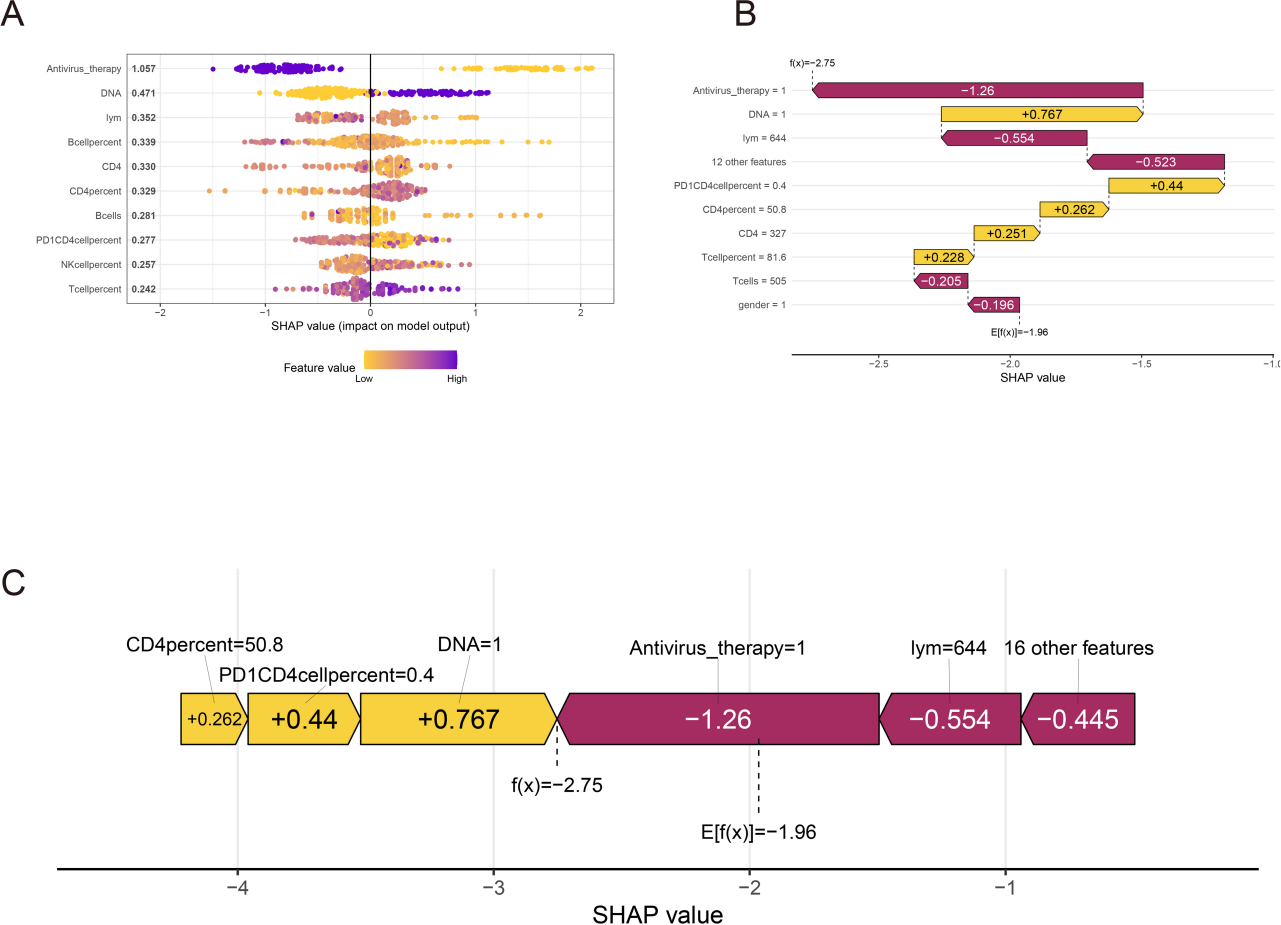
SHAP interpretation of xgboost clinical parameters. **(A)** Xgboost screening clinical parameter shape value importance ranking. **(B, C)** The shap value represents the predictive characteristics of each clinical parameter and the contribution of each parameter to the occurrence of immune-related adverse events. f(x) represents the probability prediction value, red indicates low risk, and yellow indicates high risk.

Randomforest and XGBoost regression are commonly used tree-based machine learning methods for predicting variable importance. In this study, to identify effective predictors of IRAEs (irAEs), we employed both random forest (package_version 4.7) and XGBoost (package_version 2.1.3) models to evaluate the importance of relevant variables. Using random forest analysis, we selected the top 10 variables based on importance rankings and illustrated the model’s error rate (**Figures 2A–C**). After constructing the XGBoost model, we similarly extracted and ranked the top 10 variables based on importance (**Figure 2D**). Next, we took the intersection of the variables identified by lasso regression, random forest, and XGBoost, and visualized the results. Notably, only two variables were consistently predicted by all three models: HBV DNA and antiviral therapy (**Figure 3A**). To further validate the reliability of these models, we applied a ten-fold cross-validation method. All three models demonstrated high accuracy (lasso AUC = 0.864, random forest AUC = 0.961, XGBoost AUC = 0.903) (**Figure 4A**). The precision-recall (PR) curves also showed satisfactory precision and recall rates for all models (lasso PR AUC = 0.607, random forest PR AUC = 0.892, XGBoost PR AUC = 0.768) (**Figure 4B**).

### SHAP to xgboost model importance explained

**Figure 5:**
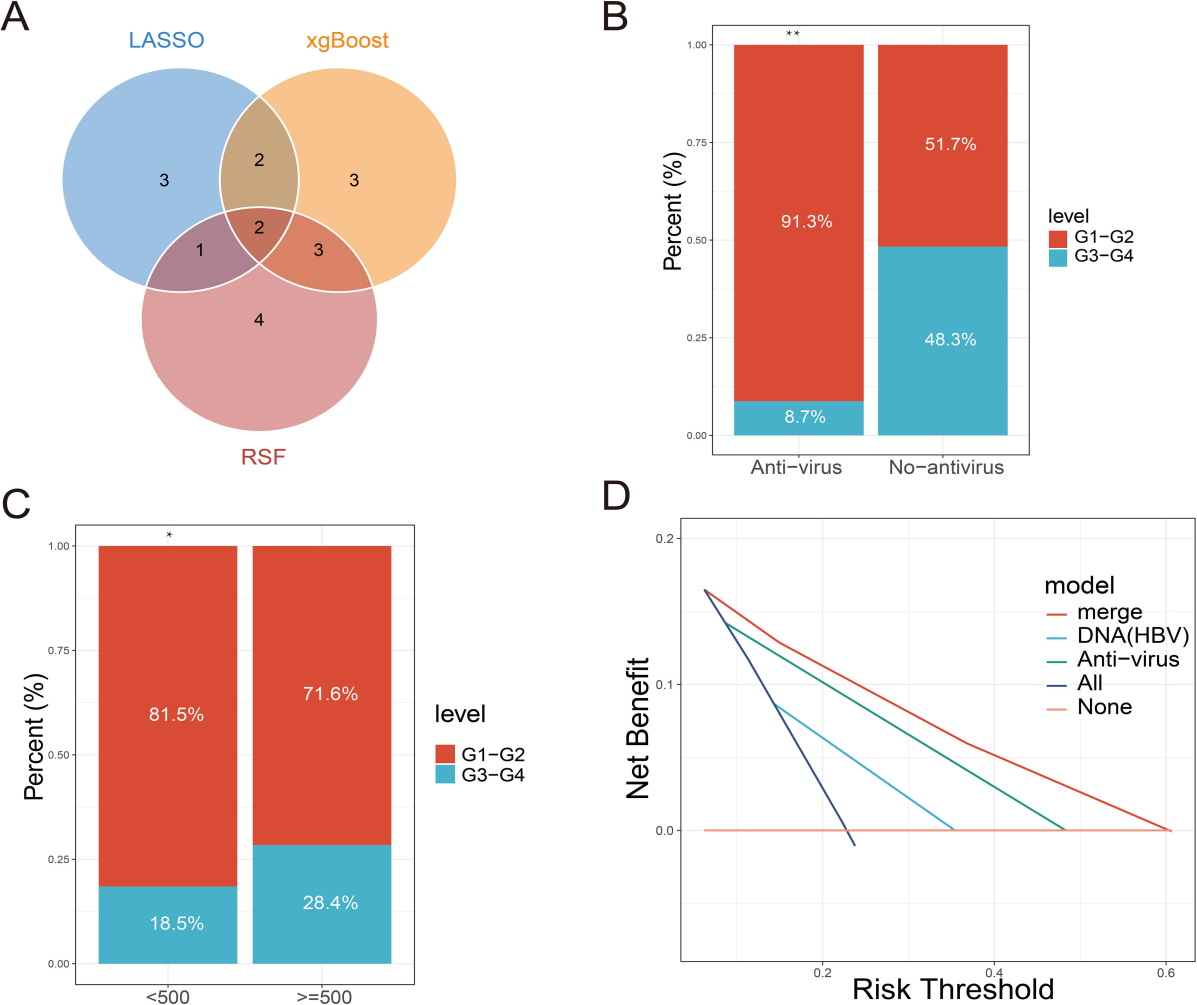
Relationship between screening indicators and clinical events based on machine learning. **(A)** Intersection of multiple machine learning screening indicators. **(B)** Comparison of the proportion of immune-related adverse events at all levels between the antiviral treatment and non-antiviral treatment groups. **(C)** Comparison of the proportion of immune-related adverse events at each level in patients with different HBV DNA copies. **(D)** DCA curves for predicting immune-related adverse events by antiviral therapy, HBV DNA alone or in combination.

To further provide a clear and intuitive explanation of the selected variables, SHAP (Shapley Additive explanations) values were utilized to elucidate the contribution of the variables in predicting IRAEs (irAEs) within the models. **Figure 5A** illustrates the SHAP values of the top 10 most important variables in the model. In the plot, blue represents high-risk factors, while yellow indicates low-risk factors. Antiviral therapy was identified as a low-risk factor for irAE occurrence, whereas high levels of HBV DNA were found to be a high-risk factor for irAEs. Other variables, such as the percentage and absolute counts of immune cells, including CD4+ T cells, NK cells, and B cells, were not significantly predictive of irAEs. **Figure 5B** ranks the SHAP absolute values of the top 10 variables identified by the XGBoost model, with the x-axis indicating the importance of the variables in predicting irAEs. Additionally, we enhanced the interpretability of the XGBoost prediction model using a typical SHAP model (**Figure 5C**). In this model, antiviral therapy had the lowest score, indicating its role as a protective factor against irAEs, while HBV DNA had the highest score, reinforcing the notion that uncontrolled HBV DNA levels or significant HBV reactivation is a strong driver of irAE development. This finding further supports that antiviral therapy can effectively mitigate the risk of irAEs.

### Antiviral therapy predicts irAEs

By constructing multiple machine learning models, this study identified antiviral therapy and low HBV DNA copy numbers as effective predictors of IRAEs (irAEs). Subsequently, we compared the incidence of irAEs between two groups: patients receiving antiviral therapy and those who were not, as well as among patients with different levels of HBV DNA copies. The results revealed that irAEs in patients receiving antiviral therapy were primarily concentrated in Grades 1–2, while patients not receiving antiviral therapy predominantly experienced Grade 3–4 irAEs (**Figure 3B**). This indicates that antiviral therapy effectively reduces the occurrence of severe irAEs. Among patients with low HBV DNA copy numbers, the proportion of Grade 3–4 irAEs was significantly lower, following a similar trend to that observed in patients receiving antiviral therapy (**Figure 3C**). We hypothesize that antiviral therapy either effectively controls HBV DNA replication or inhibits the reactivation of HBV DNA triggered by immune checkpoint inhibitors, thereby reducing the occurrence of irAEs. DCA (Decision Curve Analysis) further demonstrated that the predictive performance of antiviral therapy for irAE occurrence outperformed that of HBV DNA copy number alone. This suggests that antiviral therapy not only suppresses HBV DNA replication but also modulates immune factors or immune cells involved in irAE development. However, the combined prediction of both factors yielded the best predictive performance (**Figure 3D**).

### Relationship between antiviral therapy and immune cells

**Figure 6:**
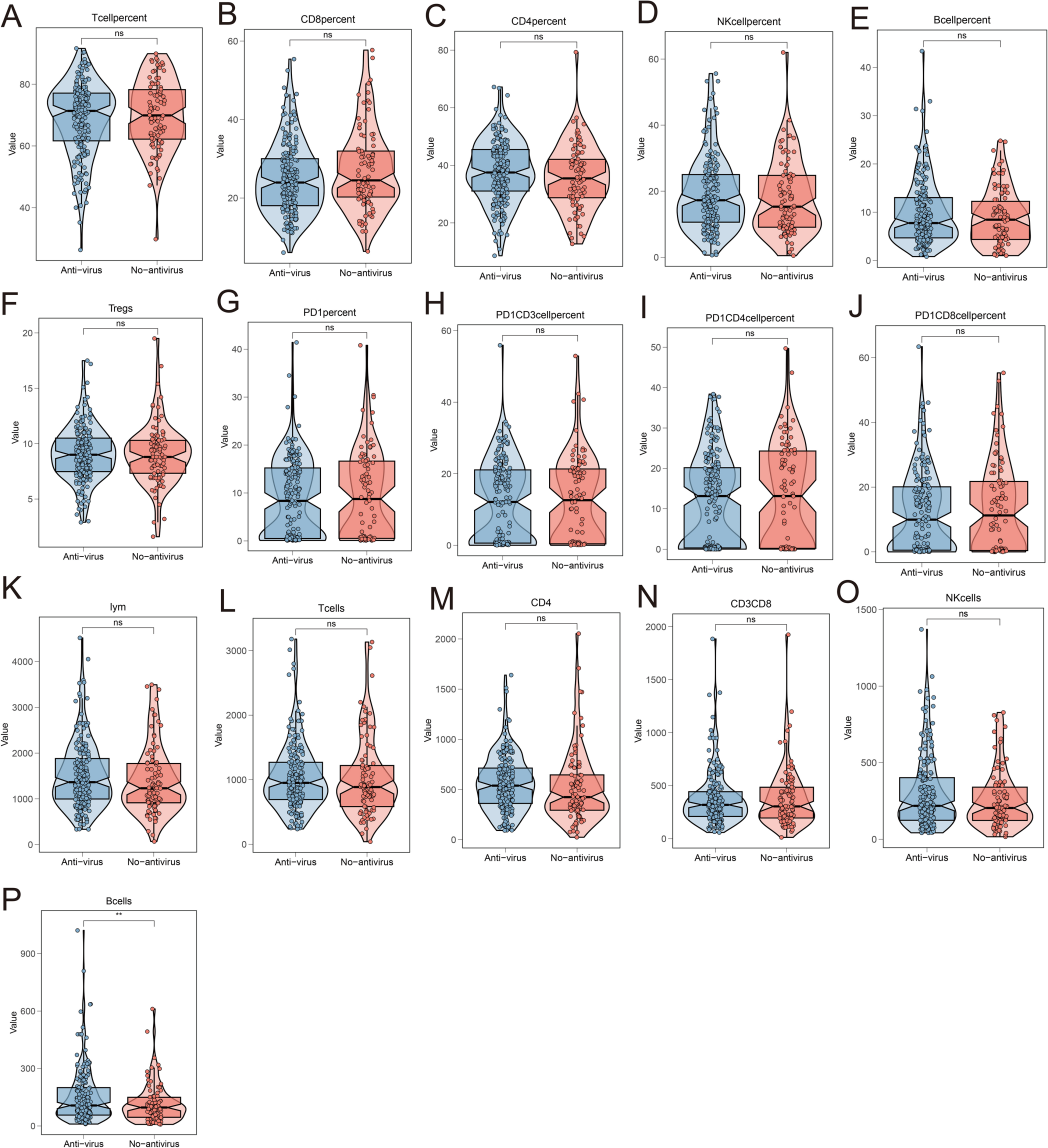
Analysis of the relationship between antiviral treatment and circulating immune cell levels. The changes in the levels of circulating immune cells in the peripheral blood of patients receiving antiviral treatment and not receiving antiviral treatment included the percentage of T cells **(A)**, the percentage of CD8^+^T cells **(B)**, the percentage of CD4^+^T cells **(C)**, the percentage of NK cells **(D)**, the percentage of B cells **(E)**, the percentage of Tregs cells **(F)**, the percentage of PD-1^+^ cells **(G)**, the percentage of PD-1^+^CD3^+^ lymphocytes **(H)**, the percentage of PD-1^+^CD4^+^T cells **(I)**, the percentage of PD-1^+^CD8^+^T cells **(J)**, the total number of lymphocytes **(K)**, the total number of T cells **(L)**, the absolute value of CD4 **(M)**, the absolute value of CD8 **(N)**, the absolute value of NK **(O)**, and the absolute value of B cells **(P)**.

The preceding results indicated that antiviral therapy effectively reduces the occurrence of irAEs and, when combined with low HBV DNA copy numbers, serves as a reliable predictor of irAE development. To further explore the relationship between antiviral therapy and immune cells, we analyzed the differences in peripheral blood immune cell levels between patients receiving and not receiving antiviral therapy. The findings revealed a significant increase in the absolute number of B cells in patients undergoing antiviral treatment, whereas no notable changes were observed in the levels of other immune cells (**Figures 6A–P**).

**Figure 7:**
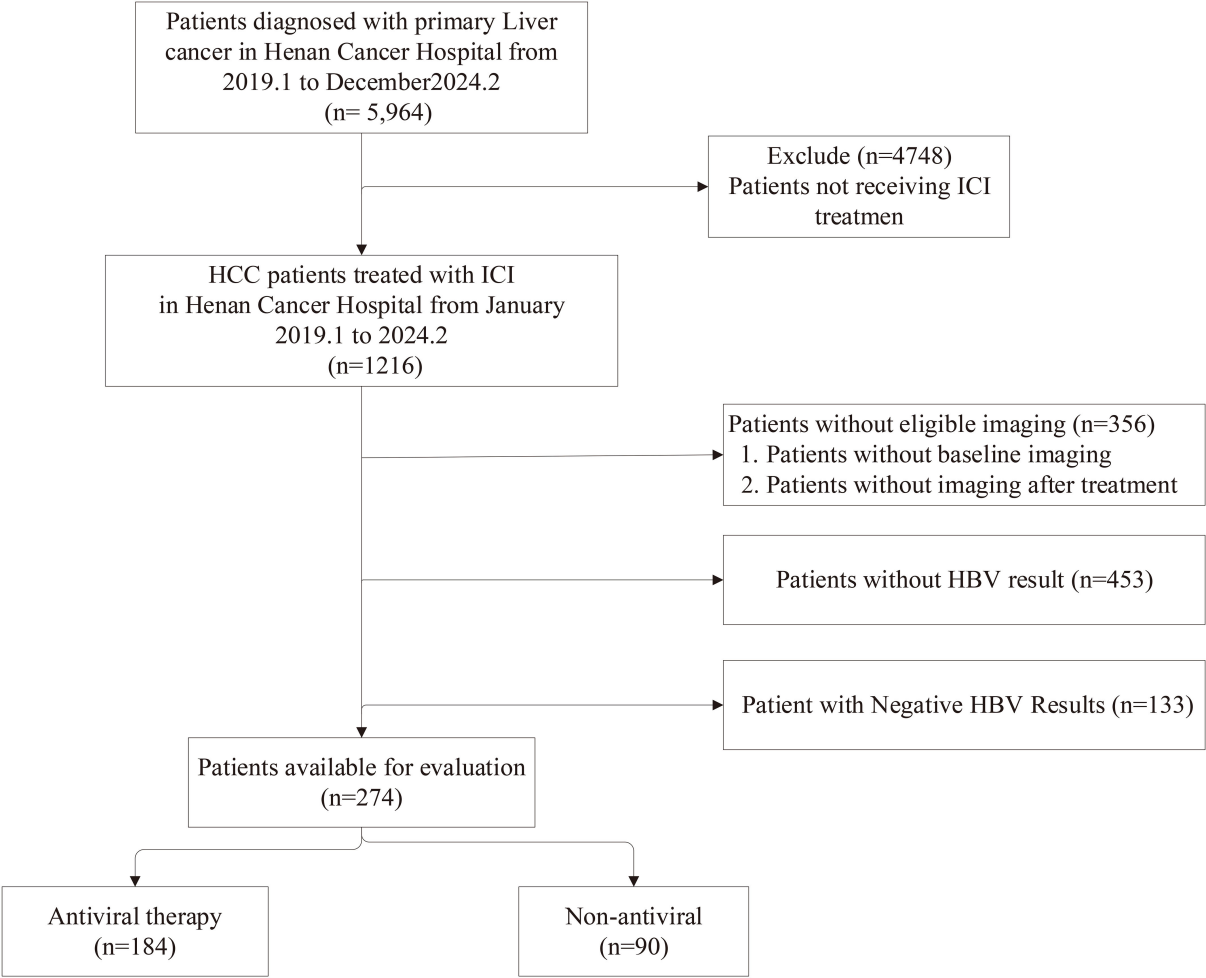
Sample collection flow chart.

## Discussion

This study evaluated the safety and efficacy of PD-1 immune checkpoint inhibitors in the treatment of HBV-associated hepatocellular carcinoma. Using multiple machine learning models, we identified biomarkers that can predict IRAEs (irAEs).

Among HBV positive hepatocellular carcinoma patients received treatment with anti-PD-1, 60 patients experienced grade 3-4 IRAEs (irAEs). Of these, 16 patients were undergoing anti-viral. When comparing patients receiving anti-viral to those who were not, irAEs in patients treated with antiviral therapy were predominantly grade 1-2, whereas those without antiviral treatment mainly exhibited grade 3-4 irAEs. Additionally, the analysis revealed that patients with low HBV DNA copy numbers or lower viral activity primarily experienced grade 1-2 irAEs, while those with high HBV DNA copy numbers or reactivated HBV exhibited more frequent grade 3-4 irAEs.

Machine learning is a mathematical discipline that primarily focuses on enabling computers to learn from data (36, 37). In medical research, machine learning models can process data using supervised or unsupervised methods to develop models that identify effective clinical predictors. These models have been applied in areas such as drug response prediction, surgical readmission risk, and patient prognosis (38–41). Common techniques for building clinical machine learning models include LASSO regression, random forest, and XGBoost, which have already been widely used for the selection and prediction of various clinical indicators. For example, machine learning has been used to predict lung cancer recurrence and assess the risk of postoperative thrombosis (42, 43). The combined use of multiple machine-learning models can further enhance the precision of these predictions. Previous studies have utilized various machine learning methods in tandem to predict clinically relevant indicators, demonstrating the reliability and improved performance of these integrated approaches (44, 45).

Here, we first employed lasso-regression to analyze the included clinical indicators with the aim of identifying biomarkers capable of predicting the coming up and severity of irAEs. The results indicated that factors such as age, gender, HBV DNA copy number, antiviral treatment, absolute B cell count, and CD4 T cell percentage were associated with irAE occurrence. Subsequent uni/multivariate logistic regression analyses revealed that HBV DNA copy number, antiviral treatment, and PD1CD3 lymphocytes may serve as independent risk factors for predicting the occurrence of irAEs. According to existing reports, irAEs arise due to ICIs not only blocking immune targets but also activating the immune system, which can trigger autoimmune responses. This activation leads to the release of related effector molecules, which in turn conduce to the development of irAEs (46, 47). HBV-virus infection can recruit a large number of inflammatory factors within the liver, which in turn attract regulatory immune cells (48). These regulatory immune cells are involved in the occurrence of irAEs (46), aligning with our predicted results. Antiviral therapy is currently the mainstay treatment for HBV infection. It has the potential to reverse T cell exhaustion and maintain immune tolerance (49), which may be the underlying reason why antiviral treatment can mitigate the occurrence of irAEs.

CD8^+^T cells make a crucial role in viral clearance and are also key components of anti-tumor immunity (50, 51). However, in patients with chronic HBV infection, CD8^+^T cells exhibit signs of exhaustion, with elevated expression of inhibitory checkpoints like PD-1, along with reduced cytotoxic and killing functions. PD-1 inhibitors, by blocking-up the PD-1/PD-L1 singling pathway, can recover CD8^+^T cell functionality and assist in clearing HBV. However, studies have shown that PD-1 inhibitors may lead to the reactivation of HBV DNA in patients with HBV-related liver cancer (52), suggesting that high HBV DNA levels are a significant risk factor for irAEs. This finding aligns with our prediction that antiviral therapy can effectively reduce the incidence of irAEs.

Additionally, this retrospective study revealed that antiviral therapy can modulate immune cell activity. In HBV positive hepatocellular carcinoma patients receiving anti-viral treatment, there was an evidently increase in the absolute count of circulating B cells, whereas changes in other circulating immune cells were not as pronounced. Previous reports have also identified a reduction in circulating cells as being closely related with the occurrence of severe irAEs (53). However, the underlying mechanisms warrant further investigation. Finally, we conducted Decision Curve Analysis (DCA) to compare the accuracy of predicting irAEs between antiviral treatment and HBV DNA copy.

B cells, as an important component of humoral immunity, participate in the process of clearing viruses in the body. Studies have found that when B cells are cleared by rituximab, HBV replication will be reactivated, leading to aggravated HBV infection (54, 55). In addition, HBVAg-specific B cells can highly express genes for cross-presenting dendritic cell recruitment (XCL1 and CD40LG) and innate immunity (MYD88, IFNA1/13, IFNa2 and IFNB1) to assist humoral immunity in resisting HBV infection (56). In the study, we found that after receiving antiviral treatment, the absolute number of B cells circulating in the patient’s peripheral blood increased, which may be due to the increased release of B cells induced by antiviral treatment, or it may be related to the accelerated promotion of B cells.

The novelty of this study lies in the development of an AI model specifically designed for predicting irAEs in HBV-positive liver cancer patients. This study utilized three machine learning algorithms, incorporating ten-fold cross-validation and bootstrapping for internal validation. Moreover, the comprehensive analysis of clinical indicators based on various machine learning models enhances the precision of the predictions. Nonetheless, this study has inherent limitations due to the restricted sample size. Firstly, it is a retrospective analysis based on clinical treatment data. Secondly, the study’s dataset is limited to patients from a specific geographic region, which may affect the generalizability to multi-regional populations. Finally, although the internal validation of the data confirms the reliability of the predictive model, extensive prospective data are required to further evaluate its applicability.

## Conclusion

In summary, our study developed a novel predictive model using three machine learning algorithms to forecast irAEs in HBV-positive liver cancer patients receiving immune checkpoint inhibitors. Among these, the RSF model demonstrated the best predictive performance. This provides theoretical and data support for clinicians to implement early intervention measures to prevent IRAEs.

## Data Availability

The original contributions presented in the study are included in the article/**Supplementary Material**. Further inquiries can be directed to the corresponding author.
