# Supplementary material for: Antiviral therapy can effectively suppress irAEs in HBV positive hepatocellular carcinoma treated with ICIs: validation based on multi machine learning
Source: Front Immunol. 2025 Jan 27;15:1516524. doi: 10.3389/fimmu.2024.1516524 (PMC11807960; doi:10.3389/fimmu.2024.1516524)
Supplement: Supplementary file 1 [file DataSheet1.zip › Table S1.DOCX]

Supplement table 1. Clinical baseline information of patients with HBV-related liver cancer

|  | level | Overall |
| --- | --- | --- |
| n |  | 274 |
| level (%) | G1-G2 | 214 (78.1) |
|  | G3-G4 | 60 (21.9) |
| gender (%) | female | 83 (30.3) |
|  | male | 191 (69.7) |
| age (%) | <60 | 155 (56.6) |
|  | >=60 | 119 (43.4) |
| DNA (%) | <500 | 177 (64.6) |
|  | >=500 | 97 (35.4) |
| alcohol (%) | No | 138 (50.4) |
|  | Yes | 136 (49.6) |
| Antivirus_therapy (%) | Anti-virus | 184 (67.2) |
|  | No-antivirus | 90 (32.8) |
| Surgery (%) | No | 188 (68.6) |
|  | Yes | 86 (31.4) |
| Interventional_therapy (%) | No | 75 (27.4) |
|  | Yes | 199 (72.6) |
| Radiotherapy (%) | No | 227 (82.8) |
|  | Yes | 47 (17.2) |
| Tcellpercent (mean (SD)) |  | 69.15 (11.71) |
| CD8percent (mean (SD)) |  | 25.71 (9.60) |
| CD4percent (mean (SD)) |  | 37.08 (10.98) |
| NKcellpercent (mean (SD)) |  | 18.56 (11.13) |
| Bcellpercent (mean (SD)) |  | 9.52 (6.48) |
| Tregs (mean (SD)) |  | 9.08 (2.53) |
| PD1percent (mean (SD)) |  | 8.95 (8.58) |
| PD1CD3cellpercent (mean (SD)) |  | 12.35 (11.51) |
| PD1CD4cellpercent (mean (SD)) |  | 12.49 (11.84) |
| PD1CD8cellpercent (mean (SD)) |  | 12.64 (13.11) |
| lym (mean (SD)) |  | 1472.05 (742.37) |
| Tcells (mean (SD)) |  | 1030.05 (548.01) |
| CD4 (mean (SD)) |  | 547.97 (310.99) |
| CD3CD8 (mean (SD)) |  | 377.06 (264.25) |
| NKcells (mean (SD)) |  | 285.41 (224.30) |
| Bcells (mean (SD)) |  | 143.91 (135.23) |
| AFP (%) | <400 | 164 (59.9) |
|  | >=400 | 52 (19.0) |
|  | >400 | 58 (21.2) |
| TB (mean (SD)) |  | 23.42 (23.03) |
| Albumin (mean (SD)) |  | 40.84 (6.01) |
| ALBI (mean (SD)) |  | -2.61 (0.53) |
| ALBIscore (mean (SD)) |  | 1.52 (0.54) |
| ECOG (mean (SD)) |  | 1.01 (0.60) |
| ChildPugh (%) | A | 218 (79.6) |
|  | B | 56 (20.4) |
| BCLC (%) | A | 25 ( 9.1) |
|  | B | 78 (28.5) |
|  | C | 171 (62.4) |
| ALT (mean (SD)) |  | 59.18 (59.84) |
| AST (mean (SD)) |  | 72.85 (84.49) |
| Livercirrhosis (%) | No | 95 (34.7) |
|  | Yes | 179 (65.3) |
| Vascular invasion (%) | No | 199 (72.6) |
|  | Yes | 75 (27.4) |
| PD-1inhibitor (%) | camrelizumab | 190 (69.3) |
|  | camrelizumab+Sintilimab | 6 ( 2.2) |
|  | camrelizumab+Tislelizumab | 4 ( 1.5) |
|  | Nivolumab | 5 ( 1.8) |
|  | Pembrolizumab | 1 ( 0.4) |
|  | Pembrolizumab+Toripalimab | 1 ( 0.4) |
|  | Sintilimab | 33 (12.0) |
|  | Tislelizumab | 32 (11.7) |
|  | Toripalimab+Sintilimab | 2 ( 0.7) |
| outcome (%) | CD | 1 ( 0.4) |
|  | CR | 7 ( 2.6) |
|  | PD | 52 (19.0) |
|  | PR | 86 (31.4) |
|  | SD | 128 (46.7) |
