# Supplementary material for: Antiviral therapy can effectively suppress irAEs in HBV positive hepatocellular carcinoma treated with ICIs: validation based on multi machine learning
Source: Front Immunol. 2025 Jan 27;15:1516524. doi: 10.3389/fimmu.2024.1516524 (PMC11807960; doi:10.3389/fimmu.2024.1516524)
Supplement: Supplementary file 1 [file DataSheet1.zip › Table S2.DOCX]

Supplement table 2. Clinical baseline information of patients with different immunotherapy responses

| name | levels | PD/SD (N=181) | PR/CR (N=93) | p |
| --- | --- | --- | --- | --- |
| level | G1-G2 | 138 (76.2%) | 76 (81.7%) | .377 |
|  | G3-G4 | 43 (23.8%) | 17 (18.3%) |  |
| gender | female | 56 (30.9%) | 27 (29%) | .852 |
|  | male | 125 (69.1%) | 66 (71%) |  |
| age | <60 | 106 (58.6%) | 49 (52.7%) | .423 |
|  | >=60 | 75 (41.4%) | 44 (47.3%) |  |
| DNA | <500 | 110 (60.8%) | 67 (72%) | .087 |
|  | >=500 | 71 (39.2%) | 26 (28%) |  |
| alcohol | No | 90 (49.7%) | 48 (51.6%) | .866 |
|  | Yes | 91 (50.3%) | 45 (48.4%) |  |
| Antivirus_therapy | Anti-virus | 112 (61.9%) | 72 (77.4%) | .014 |
|  | No-antivirus | 69 (38.1%) | 21 (22.6%) |  |
| Tcellpercent | Mean ± SD | 69.6 ± 11.8 | 68.2 ± 11.5 | .363 |
| CD8percent | Mean ± SD | 26.2 ± 10.1 | 24.8 ± 8.5 | .279 |
| CD4percent | Mean ± SD | 36.8 ± 11.1 | 37.6 ± 10.8 | .606 |
| NKcellpercent | Mean ± SD | 18.0 ± 10.8 | 19.7 ± 11.8 | .212 |
| Bcellpercent | Mean ± SD | 9.6 ± 6.4 | 9.4 ± 6.6 | .824 |
| Tregs | Mean ± SD | 9.0 ± 2.6 | 9.3 ± 2.3 | .429 |
| PD1percent | Mean ± SD | 8.7 ± 8.2 | 9.4 ± 9.3 | .527 |
| PD1CD3cellpercent | Mean ± SD | 11.9 ± 11.3 | 13.1 ± 11.8 | .423 |
| PD1CD4cellpercent | Mean ± SD | 12.1 ± 11.7 | 13.3 ± 12.1 | .429 |
| PD1CD8cellpercent | Mean ± SD | 12.3 ± 13.0 | 13.3 ± 13.4 | .527 |
| lym | Mean ± SD | 1489.8 ± 787.5 | 1437.6 ± 648.1 | .559 |
| Tcells | Mean ± SD | 1054.5 ± 593.9 | 982.4 ± 444.6 | .260 |
| CD4 | Mean ± SD | 558.8 ± 339.6 | 526.9 ± 246.3 | .376 |
| CD3CD8 | Mean ± SD | 386.2 ± 282.8 | 359.3 ± 224.3 | .392 |
| NKcells | Mean ± SD | 277.1 ± 219.1 | 301.5 ± 234.4 | .395 |
| Bcells | Mean ± SD | 146.1 ± 139.3 | 139.7 ± 127.6 | .714 |
| PD-1inhibitor | camrelizumab | 124 (68.5%) | 66 (71%) | .759 |
|  | camrelizumab+Sintilimab | 5 (2.8%) | 1 (1.1%) |  |
|  | camrelizumab+Tislelizumab | 2 (1.1%) | 2 (2.2%) |  |
|  | Nivolumab | 3 (1.7%) | 2 (2.2%) |  |
|  | Pembrolizumab | 0 (0%) | 1 (1.1%) |  |
|  | Pembrolizumab+Toripalimab | 1 (0.6%) | 0 (0%) |  |
|  | Sintilimab | 22 (12.2%) | 11 (11.8%) |  |
|  | Tislelizumab | 22 (12.2%) | 10 (10.8%) |  |
|  | Toripalimab+Sintilimab | 2 (1.1%) | 0 (0%) |  |
