# Supplementary material for: Antiviral therapy can effectively suppress irAEs in HBV positive hepatocellular carcinoma treated with ICIs: validation based on multi machine learning
Source: Front Immunol. 2025 Jan 27;15:1516524. doi: 10.3389/fimmu.2024.1516524 (PMC11807960; doi:10.3389/fimmu.2024.1516524)
Supplement: Supplementary file 1 [file DataSheet1.zip › Table S3.DOCX]

Supplementary table3. Characteristics of the Immune-Related Adverse Events(n=274)

| **Adverse events** | **Grade 1 (n, %)** | **Grade 2 (n, %)** | **Grade3 or 4 (n, %)** |
| --- | --- | --- | --- |
| Pneumonitis | 8(2.9) | 2(0.7) | 4(1.4) |
| Skin | 16(5.8) | 4(1.4) | 12(4.4) |
| Immune colitis | 9(3.3) | 2(0.7) | 4(1.4) |
| Low fever Hypothyroidism | 21(7.7) | 16(5.8) | 13(4.7) |
| Hypoproteinemia Vomiting | 9(3.3) | 7(2.6) | 0(0) |
| Bellyache | 8(2.9) | 12(4.4) | 0(0) |
| Weak | 16(5.8) | 3(1.1) | 0(0) |
| Diarrhea | 8(2.9) | 2(0.7) | 0(0) |
| Hypertension | 7(2.6) | 11(4.0) | 4(1.4) |
| Bone marrow suppression | 11(4.0) | 2(0.7) | 8(2.9) |
| Hyperaldosteronism | 12(4.4) | 6(2.2) | 114.0) |
| Liver function injury | 16(5.8) | 0(0) | 8(2.9) |
| Sum | 147(53.7) | 67(24.5) | 60(21.8) |

Supplementary table4. Efficacy in patients receiving antiviral therapy (n=184)

| **Variable** | **Antiviral-drugs** | | | | |
| --- | --- | --- | --- | --- | --- |
|  | **Adefovir ester** | **entecavir** | **tenofovir** | **Tenofovir disoproxil** | **P value** |
| **Response** 0.52 | | | | | |
| PR/CR | 20(10.9) | 18(9.8) | 22(11.9) | 21(11.4) |  |
| SD | 21(11.4) | 15(8.2) | 17(9.2) | 11(6.0) |  |
| PD | 19(10.3) | 16(3.3) | 3(1.6) | 1(0.5) |  |
